# Correction to “Social setting interacts with hyper dopamine to boost the stimulant effect of ethanol”

**DOI:** 10.1111/adb.13432

**Published:** 2024-08-19

Murillo Gonzalez DJ , Hernandez Granados BA , Sabandal PR , Han K‐A . Social setting interacts with hyper dopamine to boost the stimulant effect of ethanol. Addiction Biology. 2024;29(6):e13420, doi:10.1111/adb.13420.38898729 PMC11187408

The footnote, “Dilean J. Murillo Gonzalez and Bryan A. Hernandez Granados contributed equally to this work”, is incorrect and should be removed. Dilean J. Murillo Gonzalez is the sole first author.

There are three institutions listed in the affiliations, which is incorrect. All works reported in the paper were done entirely at the “Department of Biological Sciences, The University of Texas at El Paso, El Paso, TX, USA”. The other institutions are the authors' present addresses as noted below.

D.M.G.’s present address: Department of Neuroscience, Baylor College of Medicine, Houston, TX, USA.

B.H.G.’s present address: Department of Biochemistry, Vanderbilt University, Nashville, TN, USA.

We apologize for these errors.

## Data Availability

The data that support the findings of this study are available from the corresponding author upon reasonable request.

